# Impact of neonatal hypoxia‐ischaemia on oligodendrocyte survival, maturation and myelinating potential

**DOI:** 10.1111/jcmm.13309

**Published:** 2017-08-07

**Authors:** Malgorzata Ziemka‐Nalecz, Justyna Janowska, Lukasz Strojek, Joanna Jaworska, Teresa Zalewska, Malgorzata Frontczak‐Baniewicz, Joanna Sypecka

**Affiliations:** ^1^ NeuroRepair Department Mossakowski Medical Research Centre Polish Academy of Sciences Warsaw Poland; ^2^ Electron Microscopy Platform Mossakowski Medical Research Centre Polish Academy of Sciences Warsaw Poland

**Keywords:** oligodendrocyte progenitor cells, myelinogenesis, hippocampal organotypic slices, oxygen and glycose deprivation, perinatal asphyxia, neonatal hypoxia‐ischaemia, gelatinases, myelin structure, electron microscopy

## Abstract

Hypoxic‐ischaemic episodes experienced at the perinatal period commonly lead to a development of neurological disabilities and cognitive impairments in neonates or later in childhood. Clinical symptoms often are associated with the observed alterations in white matter in the brains of diseased children, suggesting contribution of triggered oligodendrocyte/myelin pathology to the resulting disorders. To date, the processes initiated by perinatal asphyxia remain unclear, hampering the ability to develop preventions. To address the issue, the effects of temporal hypoxia‐ischaemia on survival, proliferation and the myelinating potential of oligodendrocytes were evaluated *ex vivo* using cultures of hippocampal organotypic slices and *in vivo* in rat model of perinatal asphyxia. The potential engagement of gelatinases in oligodendrocyte maturation was assessed as well. The results pointed to a significant decrease in the number of oligodendrocyte progenitor cells (OPCs), which is compensated for to a certain extent by the increased rate of OPC proliferation. Oligodendrocyte maturation seemed however to be significantly altered. An ultrastructural examination of selected brain regions performed several weeks after the insult showed however that the process of developing central nervous system myelination proceeds efficiently resulting in enwrapping the majority of axons in compact myelin. The increased angiogenesis in response to neonatal hypoxic‐ischaemic insult was also noticed. In conclusion, the study shows that hypoxic‐ischaemic episodes experienced during the most active period of nervous system development might be efficiently compensated for by the oligodendroglial cell response triggered by the insult. The main obstacle seems to be the inflammatory process modulating the local microenvironment.

## Introduction

Oligodendrocyte progenitor cells (OPCs) are known to play different roles in the central nervous system (CNS). Besides their crucial function which is to give rise to mature oligodendrocytes capable of myelinating the CNS, they are known to be engaged in the metabolic coupling pathway, providing the myelinated axons with energy substrates (glycogen‐derived pyruvate/lactate) 1, 2, 3. OPCs together with differentiated oligodendrocytes are also thought to maintain tissue homoeostasis and contribute to neurorestorative processes 4, 5, 6. Due to their abundance in brain parenchyma, they are able to respond readily to environmental signals triggered by CNS insult or disease. Accumulating evidence shows that OPCs proliferate, migrate and up‐regulate secretion of trophic factors contributing to neuroprotection and the long‐term repair of diseased tissue 7, 8, 9. However, their role in the locally occurring processes seems to strongly depend on the type of insult and is suspected to be detrimental in certain cases.

Oligodendrocyte maturation and their resulting ability to efficiently myelinate the developing CNS are unfortunately impaired in many congenital and acquired diseases in humans, leading to leukodysthrophic disorders. One of them results from perinatal asphyxia, which is thought to be the main cause of the long‐term neurological sequelae known as periventricular leukomalacia (PVL) 10, 11, 12. As white matter is the mainly affected structure within the injured brain 13, therefore the compelling question is whether the oligodendrocyte progenitors survive the insult and are able to maturate and to express myelin components. To date, the processes initiated by perinatal asphyxia remain unclear, hampering the ability to develop preventions.

Special attention is focused on the ability of OPCs to secrete the active form of matrix metalloproteinases type 2 (MMP‐2) and type 9 (MMP‐9), also known as gelatinases 14, 15, 16. During nervous system development, those enzymes are known to facilitate OPC migration and cell processes elongation in searching for the axons to be myelinated 17, 18, 19, 20, mainly due to their ability to remodel the extracellular matrix 18, 21, 22. However, they are also activated (especially MMP‐9) in response to pathological insults like spinal cord injury (SCI), multiple sclerosis (MS) or traumatic brain injury (TBI) 23, 24, 25, 26. A question arises whether hypoxic‐ischaemic (HI) insult triggers activation of gelatinases in oligodendroglia‐biased cells and exerts any influence on cell differentiation and their myelinogenic potential.

To address the issue, we have designed comparative studies based on *in vivo* and *vitro* models to evaluate the effects of temporal hypoxia‐ischaemia on OPC survival, proliferation and maturation during the perinatal period when gliogenesis in most pronounced.

## Materials and methods

### Organotypic hippocampal culture (OHC)

**Figure 1:**
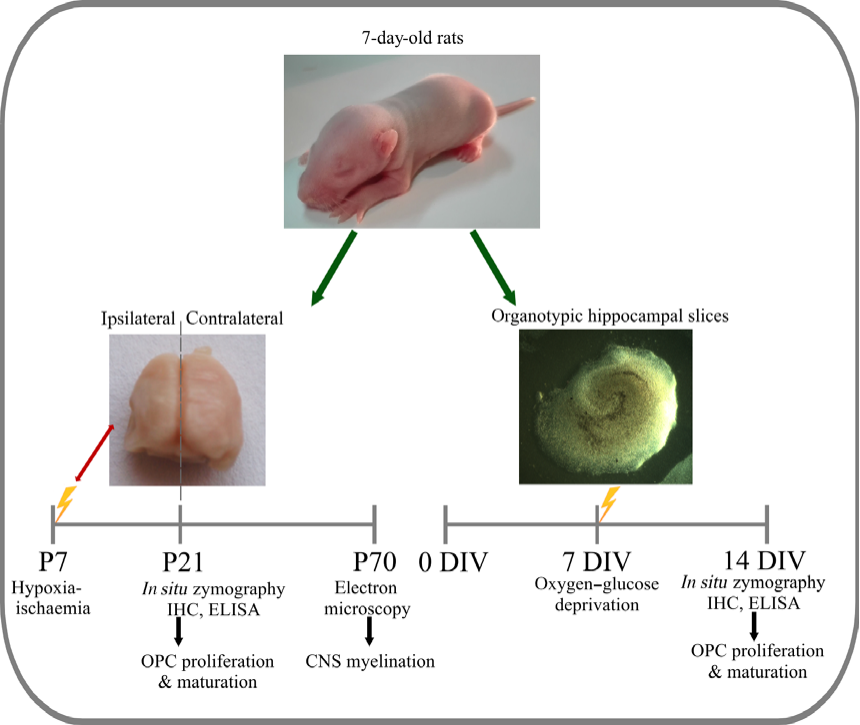
A schematic diagram of the experimental design. The parallel *in vivo* and *in vitro* studies have been established to model perinatal hypoxia‐ischaemia with aim of evaluating oligodendrocyte contribution to the resulting leukodystrophic disorders.

An *ex vivo* culture of hippocampal organotypic slices was established basing on the protocol described in detail elsewhere 8. Briefly, 7‐day‐old Wistar rats (*n* = 24) were used for the hippocampi isolation according to the procedure approved (decision no. 39/2015) by IV Local Ethics Committee on Animal Care and Use as indicated by the Ministry of Science and Higher Education. The animals were subjected to deep hypothermia on ice and decapitated. The extracted hippocampi were cut into 400‐μm‐thick slices with preserved tissue organization by Mcllwain apparatus. Placed on Millicell‐CM (EMD Millipore, Billerica, MA, USA) membranes, the slices were cultured initially in DMEM medium (Gibco) containing horse serum (25%), HBSS (25%) and additionally glucose (2 mmol/l), HEPES (5 mg/ml) and antibacterial–antimycotic solution. Starting from the 2^nd^ day *in vitro* (DIV), the serum content was gradually lowered and finally from 5^th^ DIV onwards, the slices were cultured in serum‐free conditions (DMEM+ antibiotics) up to 14 DIV (Fig. 1).

### Model of temporary oxygen–glucose deprivation

An oxygen and glucose deprivation (OGD) procedure was performed on 7^th^ DIV (Fig. 1), as previously described 27. Briefly, the glucose in the culture medium was replaced with 10 mM mannitol applied in Ringer solution saturated with 95% N_2_ and 5% CO_2_. The membranes containing hippocampal slices were inserted into an anaerobic chamber for 40 min. In this way, the culture medium was temporarily deprived of glucose and hypoxic conditions were created by eliminating oxygen from the cultures of hippocampal slices. This allowed mimicking *in vitro* the HI episode. The control slices were maintained in a glucose‐containing Ringer solution under normoxic conditions. Immediately after the OGD procedure, the slices intended for cell proliferation assay were treated for 24 hrs with 5 μM 5‐bromo‐2′‐deoxyuridine (BrdU) (Sigma‐Aldrich, Saint Louis, MO, USA). To assess the incorporation of BrdU into newly synthesized DNA of replicating cells, on 7^th^ day after the OGD the slices were fixed with 4% paraformaldehyde (PFA), washed three times with PBS and then treated on ice with 95% methanol for 10 min. After three washes with PBS, the cells were permeabilized with 2 N HCl for 10 min., RT. After neutralization of the acid with 0.1 M sodium borate for 5 min. (RT), the slices were subjected to an immunohistochemical (IHC) analysis using a panel of cell‐specific antibodies.

### Model of neonatal hypoxia‐ischaemia

Model of neonatal hypoxia‐ischaemia (HI) was performed as previously described 20. The procedure was approved by IV Local Ethics Committee on Animal Care and Use (Decision no. 83/2015). Briefly, Wistar rat pups (*n* = 24) of both sexes (12–15 g bodyweight) were anaesthetized with isoflurane (4% for induction of anaesthesia and 2.0% maintenance) on 7^th^ post‐natal day (P7) and subjected to a procedure based on the dissection of the left common carotid artery, which was either exposed (sham‐operated animals, *n* = 8) or cut between the double ligatures of silk sutures (*n* = 8). The wound was treated with lignocaine, and the animals were left in their home cages to recover for 60 min. Hypoxic‐ischaemic conditions were created by exposing the animals to 7.5% oxygen in nitrogen for 60 min. in a chamber at 35°C. As a result of the procedure, the hemisphere ipsilateral to the carotid ligation became ischaemic. The control and insulted brains were isolated on either P21 or P70, divided into ipsilateral and contralateral hemispheres and used for IHC and biochemical analyses or electronmicroscopic examination, respectively (as indicated on the scheme presented in Fig. 1).

### Preparation of coronal brain slices

Nervous tissue for the subsequent IHC analysis was obtained according to the protocol previously described 20. Generally, the deeply anesthetized animals on the 14^th^ day after HI procedure (Fig. 1) were transcardially perfused first with PBS and then with 4% PFA in 0.1 M phosphate buffer, pH 7.4 as a fixative solution. The isolated brains were post‐fixed for 3 hrs at 4°C in the same fixative solution, treated with 30% sucrose, frozen on dry ice and finally stored at −70°C. To obtain the slices of nervous tissue, the frozen brains were cut at the level of the lateral ventricle and dorsal hippocampus into 30‐μm coronal sections using a cryostat (Mikrom HM 55OP). The obtained slices were then subjected to *in situ* zymography combined with IHC analysis with specific antibodies relevant to the subsequent stages of oligodendrocyte maturation.

### *In situ* zymography and immunodetection

Gelatinase activity in differentiating oligodendrocytes both in organotypic hippocampal slices and in the brain coronal sections was visualized by performing the technique of *in situ* zymography. Accordingly, either fresh organotypic slices or fixed brain sections were treated with a reaction buffer containing 50 mg/ml of fluorescein isothiocyanate‐labelled dye‐quenched gelatin (FITC‐labelled DQ‐gelatin; Invitrogen, Thermo Fisher Scientific, MA, USA) for 3 hrs at 37°C in a humid dark chamber. Fluorescence (attributed to FITC labelling) resulting from gelatinase cleaving indicated the proteolytic activity of gelatinases. Subsequently, the hippocampal slices were fixed for 20 min. with 4% PFA. Both types of slices (*i.e*. hippocampal slices used for *in vitro* studies and coronal brain sections obtained from the *in vivo* model) were subjected to an immunocytochemical analysis to evaluate the process of oligodendrocyte differentiation. To make it possible, the following markers relevant to specific stages of oligodendrocyte maturation were applied: against chondroitin sulphate proteoglycans (NG2) for progenitor cells (1:200; Chemicon, Temecula, CA, USA), anti‐O4 (1:100; Sigma‐Aldrich) for immature cells, antigalactosylceramidase (GalC) for maturating oligodendrocytes (1:200; Chemicon), as well as against myelin basic protein (MBP) (1:100; Chemicon) and antiproteolipid protein (PLP) (1:100; Chemicon) for cells with myelinating potential. Microglial cells were detected with rat monoclonal anti‐ED1 (CD68) (1:100; AbD Serotec, Bio‐Rad, Hercules, CA, USA). Firstly, the unspecific binding of the markers used was prevented by incubating the slices in PBS containing 10% normal goat serum for 1 hr, room temperature (RT). The primary antibodies were applied over nightly at 4°C, and secondary antibodies conjugated to Alexa 546 fluorochrome (1:500; Molecular Probes, Eugene, OR, USA) were applied for 1 hr, RT. The cell nuclei were visualized by treatment with 5 μM Hoechst 33258 (Sigma‐Aldrich), for 20 min., RT, and then the slices were immersed in Fluoromount (Dako, Dako, Glostrup, Denmark). Obtained in this way slides were examined using the LSM 780/ELYRA PS.1 super‐resolution confocal system (Carl Zeiss, Jena, Germany). Double‐labelled cells (active MMPs detected by *in situ* zymography and oligodendrocyte markers visualized by IHC) were counted, and their number at subsequent stages of oligodendrogial differentiation was evaluated.

### Cell counting and statistical analysis

Counting of the cells with well‐recognizable cell bodies and nuclei was performed on a randomly selected area (606.3 × 606.3 μm) within the coronal sections of the defined brain regions and on the organotypic hippocampal slices. The number of MMPs/NG2‐positive cells was estimated in the frontal and pariental cortex in five to seven randomly selected areas of the layers IV, V and VI on each slice. Evaluation of MMPs activity in the striatum was performed in a similar way (in five to seven randomly selected areas). Amount of MMPs/NG2^+^ cells was also evaluated in the entire hippocampus (including DG area and CA1‐CA3 regions) in an average of five sections per animal. In the organotypic hippocampal slices routinely three to five areas were analysed on each of at least three slices from each of the three experiments. Cell counting was performed by manually dotting each of the analysed markers, and ImageJ 1.46 software (https://imagej.nih.gov) was used for automatic scoring of the marked cell. In certain cases (especially when estimating the MBP expression), the intensity of a fluorescence signal was additionally measured by application of ImageJ software. When counting was completed, the numbers of individual pictures were disclosed and obtained data were subjected to the statistical analysis with GraphPad PRISM 5.0 software. Comparisons between experimental groups were performed using one‐way analysis of variance (anova) with *post hoc* Bonferroni test for multiple comparisons. All the values were expressed as mean ± S.E.M. The calculated differences were marked as significant if *P < 0.05, **P < 0.01; ***P < 0.001.

### Biochemical determination of oligodendrocyte‐associated protein expression

To evaluate the contents of the oligodendrocyte‐associated proteins in both the rat brains and in the hippocampal slices, the Western blot analyses with specific antibodies were carried out, as previously described in detail 28. Accordingly, the hippocampal slices and the brain hemispheres were gently homogenized in CelLytic™ MT cell lysis buffer (Sigma‐Aldrich) supplemented with protease inhibitor cocktail (Sigma‐Aldrich). The concentration of protein was determined by the modified Lowry method, using DC Protein Assay (Bio‐Rad). Samples (50 μg protein) were ran on 10% SDS‐PAGE gels and transferred onto nitrocellulose membranes (Amersham, Sigma‐Aldrich). After blocking in 5% non‐fat milk in TBST, membranes were incubated with specific primary antibodies. Assays were performed with the use of specific antibodies against anti‐NG2 (1:200; Millipore), anti‐GalC (1:500; Millipore), anti‐MBP (1:500; Millipore), anti‐PLP (1:500; Millipore) and anti‐actin (1:500; MP Biomedicals, Santa Ana, CA, USA), respectively. Then, the membranes were incubated with secondary antibody conjugated to horseradish peroxidase (Sigma‐Aldrich). Immunoblot signals were visualized using ECL chemiluminescence kit (GE Healthcare Life Sciences, Chicago, IL, USA). To verify an equal loading of protein per line, the anti‐actin antibody was used as an internal control for each immunoblotting. Semi‐quantitative evaluation of protein levels detected by immunoblotting was performed by computer‐assisted densitometric scanning (LKB Ultrascan XL, Program GelScan).

Subsequently, with the aim of determining the amounts of major myelin‐specific proteins, the ELISA assays were performed for MBP and PLP (Abbexa, Cambridge, UK), following the supplier's instructions. The plates were read at 450 nm using a spectrophotometric plate reader FLUOstar Omega (BMG LabTech, Ortenberg, Germany). The obtained data were statistically analysed by Mann–Whitney test (two‐tailed with Gaussian approximation). All values were expressed as mean ± S.E.M. and were considered as statistically significant if *P < 0.05, **P < 0.001.

### Analysis of myelin sheath formation by electron microscopy

The efficiency of myelination after HI insult was assessed by examination of myelin ultrastructure by means of electron microscopy. At P70 (Fig. 1), rats (*n* = 8) were anaesthetized and gently perfused with fixative solution composed of 2% PFA, 2.5% glutaraldehyde and 0.1 M cacodylate buffer, pH 7.4. The selected brain regions (hippocampus, striatum, cerebral cortex, corpus callosum) were isolated and additionally were fixed overnight in the same solution and then post‐fixed in 1.5% OsO_4_ and 0.8% K_4_(FeCN)_6_ for 2 hrs. The post‐fixed brain sections were dehydrated in ethanol and propylene oxide. The analysis of the ultrathin section (60 nm) was performed using transmission electron microscopy (JEM‐1200EX; Jeol, Tokyo, Japan) with special focus on the myelin ultrastructure. Accordingly, at least one hundred of myelinated fibres in each of the analysed brain region per animal were analysed and the same amount of brain vessels was counted in context of correctly compacted myelin sheaths and bridging vessels, respectively.

## Results

### Oligodendrocyte progenitors exhibit gelatinase activity after perinatal HI

The number of OPCs expressing the active forms of metalloproteinases was determined on day 14 after the hypoxic‐ischaemic insult was applied to the 7‐day‐old rat pups. The performed analyses of the colocalization of the glia‐biased (NG2^+^) cells and active forms of MMP‐2/MMP‐9 in the selected regions of the brain coronal sections allowed us to estimate whether the gelatinase involvement in the cell differentiation had been somehow affected.

**Figure 2:**
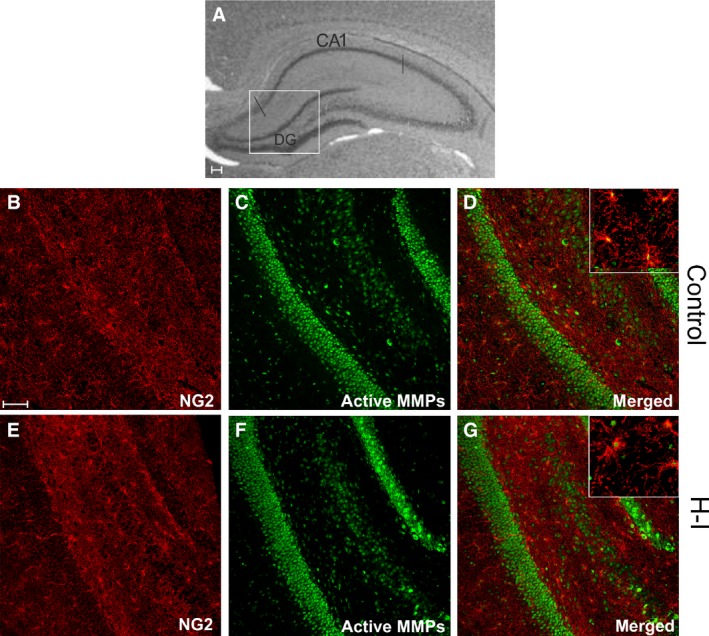
OPCs expressing NG2 marker (red) and active gelatinases (green) in rat hippocampus 14 days after H‐I; (**A**) characteristic structure of rat hippocampus with easy discernable neurogenic regions (DG and CA1); (**B**–**D**) controls; (**E**–**G**) injured animals (ipsilateral hemisphere, lower panel). Most of the gelatinase activity is localized within DG and CA1 regions. Scale bar corresponds to 100 μm.

**Figure 3:**
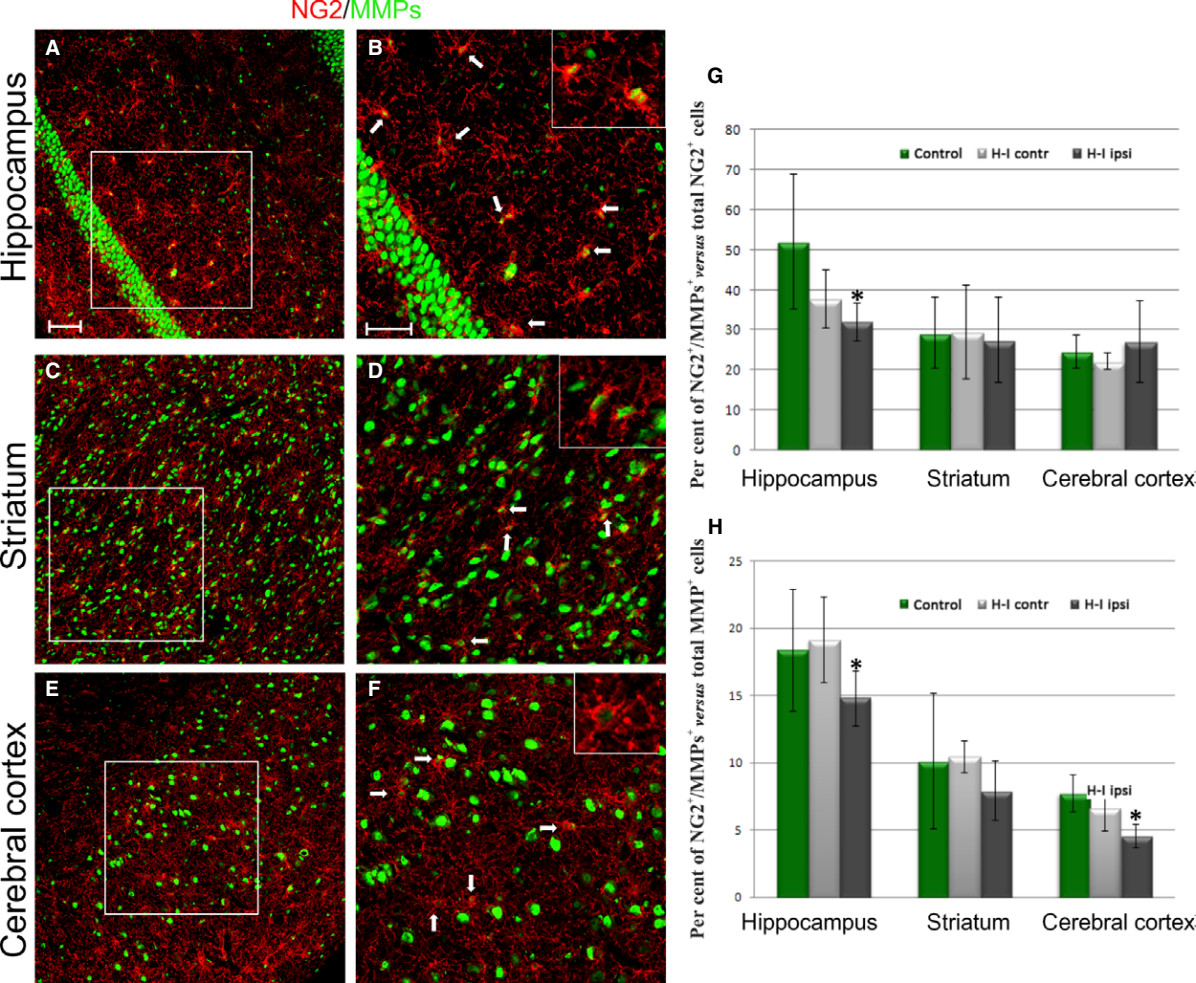
Colocalization of OPCs and active gelatinases in selected regions of the brain 14 days after H‐I. Immunohistochemical double‐labelling of OPCs: NG2 marker (red) and MMP‐2/MMP‐9 (green) in: (**A, B**) hippocampus; (**C, D**) striatum and (**E, F**) cerebral cortex. Oligodendroglial progenitors expressing active gelatinases are numerous in the examined brain regions and some of them are indicated by white arrows on the magnifications of the boxed‐in areas. Scale bar is the equivalent of 100 μm. The relative number of OPCs expressing active forms of MMP‐2/MMP‐9 calculated *versus*: (**G**) total OPC fractions in selected regions of control and hypoxic‐ischaemic brains; (**H**) total number of gelatinase‐positive cells in developing rat brains. After H‐I, the fraction of gelatinase‐expressing OPCs decreases significantly within hippocampus. Comparison of NG2^+^/MMPs^+^ cell number with the total fraction of cells expressing gelatinases in rat brain after H‐I points to a significant down‐regulation of the former in hippocampus and in cerebral cortex. The one‐way analysis of variance (anova) followed by the Bonferroni's multiple comparison (*n* = 10, number of analysed brain slices was 12 for each animal). All values were expressed as mean ± S.D.; *P < 0.05.

Within the hippocampal formation, the characteristic neurogenic regions could be distinguished and identified as dentate gyrus (DG) and *cornus ammonis* (CA) (Fig. 2A). According to the obtained data, the gelatinase activity was predominantly detected in those areas populated with cells with precursor characteristic. Furthermore, IHC double‐labelling allowed visualizing the oligodendroglial progenitors which were positive for both the NG2 marker and the active gelatinases (NG2^+^/MMPs^+^) (Fig. 2B–D). Their number however seemed to have diminished in the hippocampus of the ipsilateral hemisphere after the hypoxic‐ischaemic insult (Fig. 2E–G). A microscopic examination of the cropped areas of HI brains suggested that the evaluated cell portion, unlike in the hippocampus (Fig. 3A and B), was almost intact in both the striatum (Fig. 3C and D) and the cerebral cortex (Fig. 3E and F). The visualized OPCs characterized by MMP‐2/MMP‐9 activity were counted, and their number was calculated *versus* the total OPC fraction in different brain regions (Fig. 3G). The compared subfractions included those present in the ipsilateral (*i.e*. hypoxic‐ischaemic), the contralateral (hypoxic) and the control brain hemispheres. A statistical analysis confirmed the decreased number (from 52.03 ± 15. 83% to 31.92 ± 4.77%) of gelatinase‐positive cell within the entire pool of NG2^+^ oligodendroglial progenitors in the hippocampus of HI animals.

In context of the presented results, a question arises how large actually the NG2^+^/MMPs^+^ fraction among entire population of the cells exhibiting gelatinase activity in a developing rat brain is. To address this issue, the number of OPCs expressing gelatinases was calculated *versus* the total number of MMP‐2/MMP‐9‐positive cells in selected brain regions after the insult (Fig. 3H). The statistical analysis indicates the down‐regulated number of the cells in question in the hippocampus (from 18.36 ± 4.53% to 14.81 ± 1.39%) and in the cerebral cortex (7.72 ± 2.03% in controls comparing to 4.57 ± 0.87% in experimental animals) after hypoxic‐ischaemic insult.

### Differentiation of OPCs after HI *in vivo*

The next step of the designed study was to evaluate an advance in maturation process of the OPCs affected by hypoxic‐ischaemic insult. Therefore, the brain coronal sections were stained with anti‐GalC antibody to detect the classic marker of the differentiating oligodendrocytes. Engagement of gelatinases in the process examined *in vivo* was assessed again by the *in situ* zymography technique. Visualizing the cells in the selected regions of the brain enabled us to observe different cell morphology.

**Figure 4:**
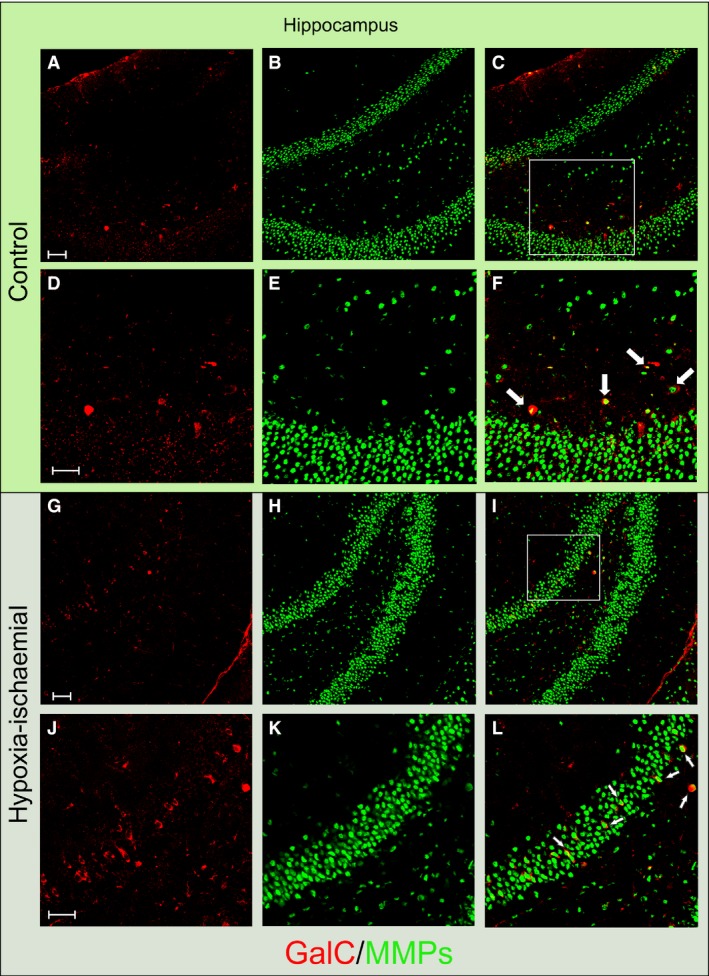
Similar number of differentiating oligodendrocytes visualized in control rat hippocampus and after hypoxic‐ischaemic episode: maturating GalC^+^ (red) and MMPs^+^ (green) cells. (**A**) GalC‐positive cells in controls; (**B**) gelatinase‐expressing cells in controls; (**C**) merge; (**D**–**F**) Enlargement of the framed region. Lower panel: hippocampus of H‐I rats 14 days after the insult: (**G**) differentiating, GalC^+^ oligodendrocytes; (**H**) cells with active gelatinases; (**I**) merge. (**J**–**L**) A magnified image of the boxed‐in area: differentiating oligodendrocytes characterized by the expression of the active gelatinases are indicated by the white arrows. Scale bar is the equivalent of 100 μm.

In hippocampus, both the control (Fig. 4A–F) and that injured by HI in the ipsilateral hemisphere of the experimental animal brain (Fig. 4G–L), the examined cells were infrequently found and had the characteristic tight shape, rarely with few short processes. No significant differences in their number between injured animals and controls were observed (20.1 ± 9.7 of GalC^+^ cells in controls *versus* 17 ± 11. 27 per 0.368 mm^2^ in experimental animals). Interestingly, the majority of maturating oligodendrocytes (81.39 ± 11.92%) expressed active gelatinases.

**Figure 5:**
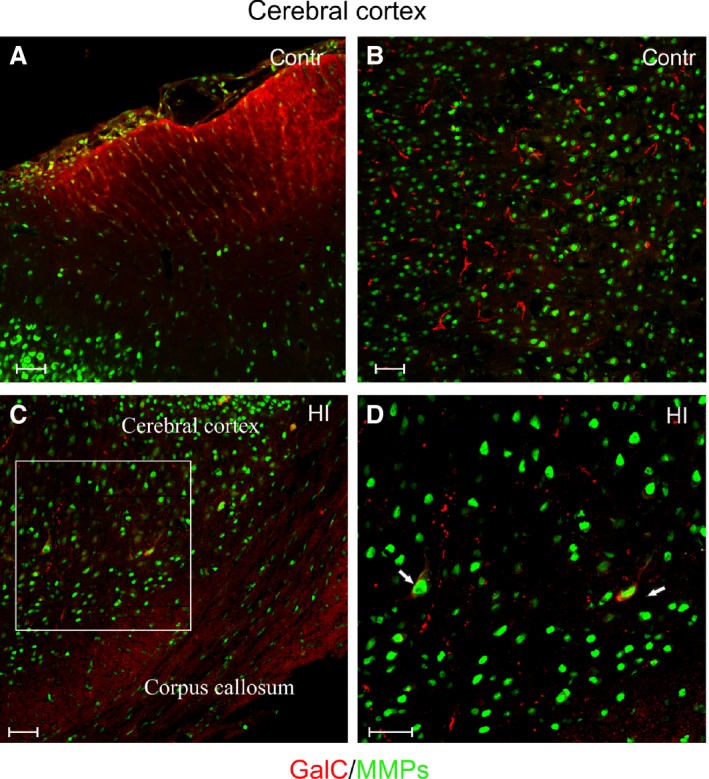
Differentiating cells in rat cerebral cortex: differentiating GalC‐expressing (red) oligodendrocytes and gelatinase‐positive cells (green). (**A**) Localization of cells in the outer region of coronal brain sections; (**B**) characteristic long oligodendroglial processes extended within the cerebral cortex. (**C**) Colocalization of oligodendrocyte cell body and gelatinases in cerebral cortex, while in corpus callosum cells are characteristically elongated. The white frame indicates the region magnified in the next picture. (**D**) Differentiating oligodendrocytes characterized by the expression of the active gelatinases (white arrows). Scale bar corresponds to 100 μm.

**Figure 6:**
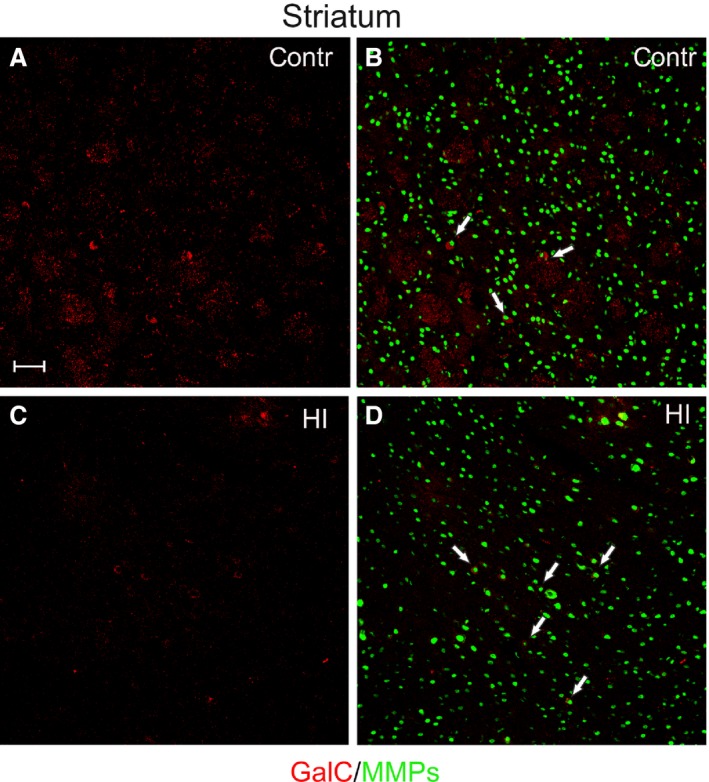
In striatum, maturating oligodendrocytes are characterized by ‘clump’ morphology (GalC, red); however, some of them express gelatinases (MMP‐2/MMP‐9, green), indicated by white arrows. (**A, B**) differentiating GalC^+^ oligodendrocytes in control rats; (**C, D**) significantly reduced number of GalC‐positive cells 14 days after hypoxic‐ischaemic insult. Scale bar corresponds to 100 μm.

In the cerebral cortex, the GalC‐positive cells were distinguished predominantly as the cells with long cellular processes (Fig. 5A and B). About 2 per cent of the stained cell expressed MMP‐2/MMP‐9 in their cell bodies (Fig. 5C and D), and there were no striking changes after HI insult in this region of the brain (34.12 ± 7.49 cells after HI *versus* 31.5 ± 13.5 in controls per 0.368 mm^2^). Conversely, in the developing striatum (Fig. 6A and B), areas of visibly reduced number of maturing GalC‐positive oligodendrocytes were found (Fig. 6C and D) and such foci were randomly scattered within this brain region.

**Figure 7:**
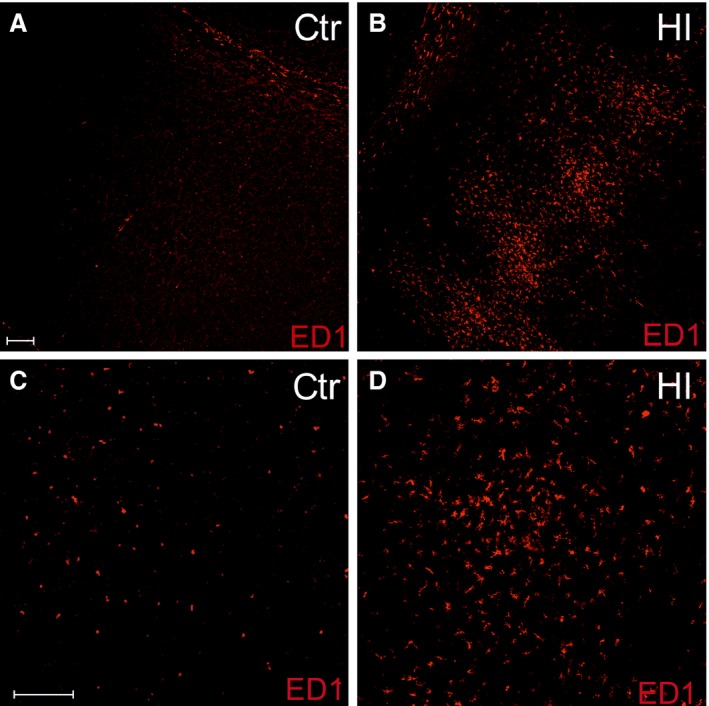
Increase in number of microglia in rat cerebral cortex 14 days after HI as indicated by cell labelling with ED1 marker (red). (**A**) Only few ED1^+^ cells could be found in the intact brain; (**B**) number of microglia significantly increases after hypoxic‐ischaemic insult; (**C**) magnification of control rat cerebral cortex with no signs of inflammation; (**D**) magnification of cerebral cortex after HI showing tremendous up‐regulation in number of cells associated with immunological response to the insult. Scale bar is the equivalent of 100 μm.

Another important finding during the IHC examination was a dramatic rise in the number of ED1^+^ microglia in the ipsilateral hemisphere after the hypoxic‐ischaemic insult: from 3.2 ± 0.57 in controls to 30.7 ± 5.8 per 0.368 mm^2^ after the injury (Fig. 7A–D). The observed 10‐fold increase in the number of cells associated with immunological response was approximately constant during the following 4 weeks. ED1^+^ cells were found predominantly within the cortex and corpus callosum of the hemisphere ipsilateral to the insult. Moreover, the cell migrated through the CC to the contralateral hemisphere; however, they do not populate any of the brain regions.

### Oligodendrocyte survival and proliferation in hippocampal organotypic slices after OGD

**Figure 8:**
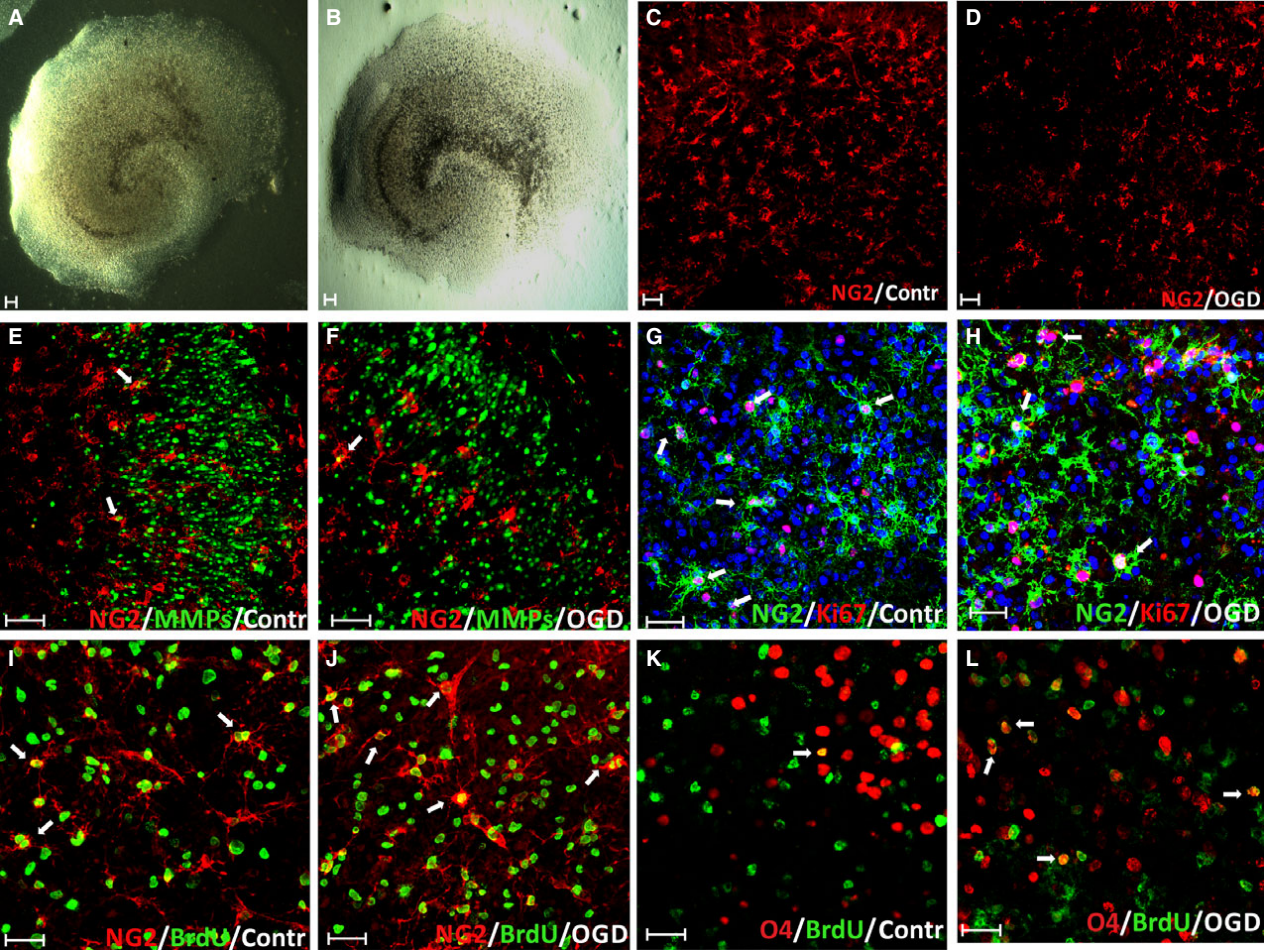
Effect of glucose and oxygen deprivation on the survival and proliferation of oligodendrocyte progenitors within organotypic hippocampal slices: (**A**) live image of control hippocampal slice; (**B**) OGD‐subjected slice with visible disintegration of its characteristic morphology; (**C**) NG2‐positive (red) progenitors in controls; (**D**) reduced number of NG2^+^ (red) progenitors after OGD insult; (**E**) oligodendrocyte progenitors (NG2^+^, red) expressing gelatinases (MMPs, green) in controls; (**F**) colocalization of NG2 marker (red) and gelatinase activity (green) in injured slices; (**G**) proliferating, Ki67^+^ (red) progenitors (NG2^+^, green) in control slices (white arrows indicate double‐labelled cells); (**H**) colocalization (white arrows) of OPCs (NG2, green) and marker of dividing cells‐Ki67 (red) 7 days after OGD, cell nuclei are stained with Hoechst 33258 (blue); (**I**) newly generated (BrdU^+^, green) OPC (NG2^+^, red) in control slices; (**J**) OPCs (NG2^+^, red) generated (BrdU^+^, green) after OGD insult; (**K**) immature (O4‐positive, red) BrdU^+^ (green) oligodendrocytes in controls; (**L**) double‐labelled immature oligodendrocytes (O4^+^, red), born‐as indicated by BrdU^+^ (green) incorporation‐after OGD procedure. Scale bar is equivalent of 50 μm.

As the most significant changes in the OPC number after hypoxic‐ischaemic insult were observed within the hippocampus (Figs 2 and 3), this region was subjected to a closer examination by applying a technique of culturing hippocampal organotypic slices. The slices are characterized by the preserved tissue architecture for a few weeks of *in vitro* culturing and therefore are thought to be a standard model of the *in vivo* processes (Fig. 8A). To make parallel studies (*in vivo versus in vitro*), 7‐day‐old rats were used again to isolate hippocampi to establish a slice culture. Subsequently, with the aim of mimicking the *ex vivo* hypoxic‐ischaemic incident, a procedure of temporarily deprive slices of glucose and oxygen was applied (Fig. 8B). Taking into consideration the fact that cells are thought to maturate more quickly when slices are being cultured *in vitro*, more advanced stages of oligodendrocyte differentiation process could be analysed.

At the very beginning of the study on the OHC, the number of NG2‐positive progenitors surviving the OGD was visualized (Fig. 8C and D), and cells expressing the active gelatinases both in controls (Fig. 8E) and in the injured slices (Fig. 8F) were stained. As revealed by OPC immunolabelling for the presence of Ki67 protein which is the marker of dividing cells, the oligodendroglial progenitors were mitotically active in the control slices (Fig. 8G), as well as in the slices subjected to the temporal deprivation of oxygen and glucose (Fig. 8H). To evaluate the influence of pathological conditions on the cell proliferation shortly after the insult, the BrdU reagent was added do the culture medium for the first 24 hrs after OGD procedure. Incorporation of the reagent into DNA of the newly generated cells was assessed by the double‐labelling procedure. It revealed the colocalization of NG2 marker and BrdU in both the controls (Fig. 8I) and in OGD‐subjected slices (Fig. 8J). The additional data gained due to double‐labelling of the slices concerned the immature, newly formed cells (BrdU^+^), which were however advanced in their differentiation process as indicated by the presence of O4 marker on the cell surface in the controls (Fig. 8K) and the injured slices (Fig. 8L).

**Figure 9:**
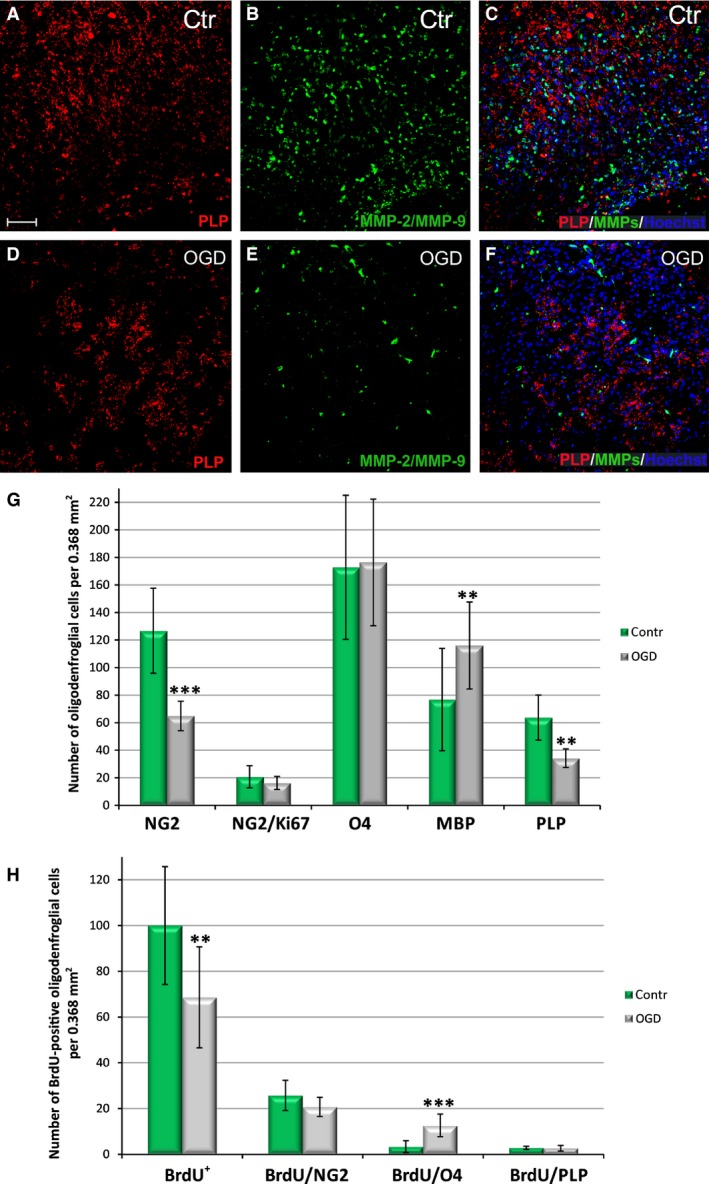
Effect of glucose and oxygen deprivation on the survival, proliferation and differentiation of rat oligodendrocyte progenitors within organotypic hippocampal slices during 7 DIV. Anti‐PLP staining (red) of mature oligodendrocytes: Colocalization with gelatinases (green) was observed neither in control slices (**A**–**C**) nor in slices injured by OGD procedure (**D**–**F**). Cell nuclei (blue) are visualized with Hoechst 33258. Scale bar is the equivalent of 100 μm. (**G**) Statistical analysis of oligodendrocyte proliferation and maturation in slices subjected to OGD procedure; (**H**) statistical analysis of the amount of the OPCs which are newly born (BrdU^+^) after the insult and progress in their maturation. The calculated differences were marked as significant if: **P < 0.01; ***P < 0.001.

Assessing the progenitor ability to maturate after a temporal deprivation of the glucose and oxygen which are the basic substrates of the cell metabolism, the major myelin proteins were detected. The expression of myelin components is considered to be the final step of oligodendrocyte differentiation associated with their potency for myelinogenesis. As visualized by IHC double‐labelling of the investigated cells, the amount of PLP‐positive cells, as well as the cells expressing gelatinases, is visibly reduced after the insult (Fig. 9A–F). Summing‐up the results obtained due to statistical analysis of the data coming from cell labelling and cell counting, the depriving the slices of oxygen and glucose for a relatively short time significantly decreases the number of OPCs in them (64.93 ± 10.7 after OGD *versus* 126.75 ± 30.84 in controls per 0.368 mm^2^). (Fig. 9G). However, as deduced from staining the newly generated OPCs with a BrdU tracker (Fig. 9H), the reduction in the cell number is partially compensated for by the cell proliferation (20.74 ± 4.21% of BrdU^+^/NG2^+^ cells after OGD compared to 25.75 ± 6.55% in controls, calculated *versus* total fraction of BrdU‐positive cells). It is worth noting that the number of the newly born OPCs during first week of culturing slices in serum‐free conditions is relatively high. Moreover, the OGD‐subjected slices were characterized by the increased percentage of newborn immature oligodendrocytes (12.62 ± 4.91% of BrdU^+^/O4^+^ cells post insult *versus* 3.37 ± 2.60% in controls) (Fig. 8E). On day 7 post‐insult, only the small fraction of existing NG2 progenitors (about 5.32 ± 2.2% in controls and 5.92 ± 2.27 after OGD) was characterized by the presence of the active forms of gelatinases. Interestingly, on 7^th^ DIV after the OGD procedure, the newly generated (BrdU^+^) and already mature (PLP‐positive) oligodendrocytes were observed in organotypic hippocampal slices and their number in experimental (2.63 ± 1.20%) and control (2.81 ± 0.71%) slices was comparable. Another interesting finding revealed a relatively numerous pool of still dividing OPCs visualized by staining cell nuclei with the Ki67 marker with no statistically significant difference between The OGD‐subjected and the control slice sand control animals (16.2 ± 4.71% dividing NG2^+^/Ki67^+^ *versus* total population of NG2‐positive cells in controls and 18.87 ± 6.99% in experimental animals).

### Influence of OGD on oligodendrocyte differentiation in hippocampal organotypic slices

**Figure 10:**
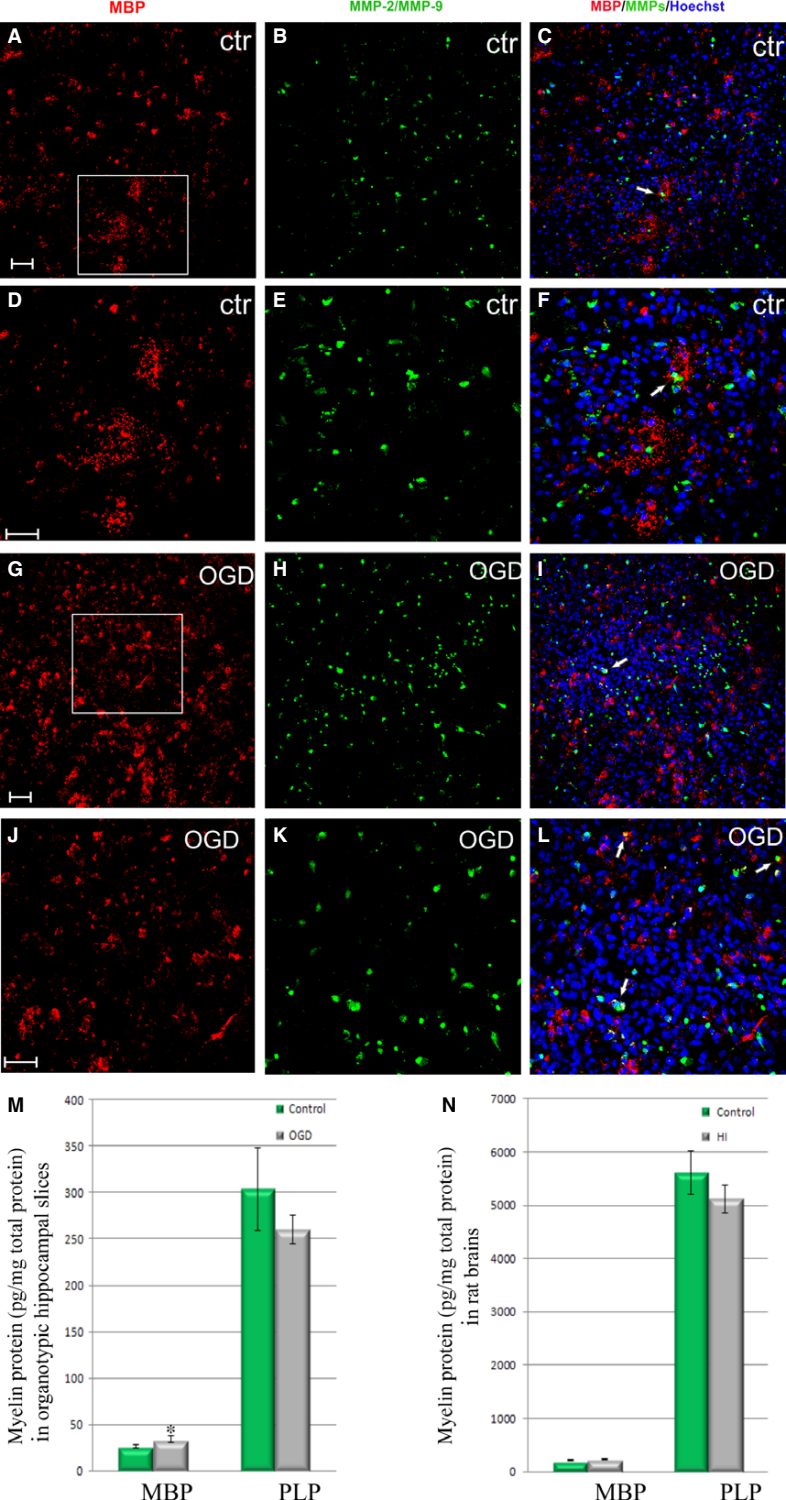
Amounts of major myelin components in both the organotypic hippocampal slices and in the rat brains 7 days after hypoxic‐ischaemic incident; (**A**–**C**) mature, control MBP‐positive (red) oligodendrocytes in organotypic hippocampal slices, solely (white arrows) expressing gelatinases (green). (**D**–**F**) enlargement of the white frame to show branched oligodendrocyte morphology; (**G**–**I**) OGD‐subjected slices; (**J**–**L**) magnification of the previous picture. Cell nuclei are stained with Hoechst 33258 (blue); Scale bar corresponds to 100 μm. (**M**) Quantitative analysis of MBP and PLP amounts in organotypic hippocampal slices; (**N**) quantitative analysis of MBP and PLP amounts in rat brains. The calculated differences were considered as significant if *P < 0.05.

Taking into consideration the fact that OGD strongly affects a number of cells at their progenitor stage, the influence of temporal shortage of glucose and oxygen on the subsequent oligodendrocyte differentiation was examined. As the final stage of oligodendrocyte maturation is their ability to express myelin building molecules, the antibodies against MBP and against PLP were used to visualize those two major components of myelin sheaths. In control slices, the cells stained either with PLP (Fig. 9A–F) or MBP (Fig. 10A–L) were usually characterized by well‐developed, branched cell processes. The statistical data obtained due to counting PLP‐positive cells revealed however a reduced (by approximately 50%) number of mature cells within the slices after the injury (34.2 ± 6.76 after OGD *versus* 63.75 ± 16.4 in controls per 0.368 mm^2^). In case of MBP, the efficiency of cell scoring was also verified by measuring the intensity of fluorescence signal. According to our observations, the MBP antigen is detected by the applied antibody during *in vitro* studies prior to PLP, although both of them are typical for mature oligodendrocytes. The statistical analysis showed an increased number of MBP^+^ cells after OGD (76.82 ± 21.37 in controls *versus* 115.06 ± 30.57 after OGD, significant at *P* < 0.05), which correlated with about a 36% increase in the intensity of the recorded fluorescence. Moreover, almost none of the matured oligodendrocytes colocalized with the MMP‐2/MMP‐9.

To verify the results obtained by application of IHC followed by detailed analysis by means of super‐resolution confocal microscopy, the biochemical techniques had been used to measure amounts of oligodendrocyte‐associated compounds in both the organotypic hippocampal slices and in rat brains subjected to hypoxic‐ischaemic insult. Accordingly, a quantitative estimation of the MBP and PLP contents in the hippocampal slices confirmed the increased amounts of MBP (26.14 ± 1.66 in controls *versus* 34.25 ± 3.32 pg/mg of total protein contents after OGD, significant at *P* < 0.05) (Fig. 10M). Amounts PLP showed tendency to decrease after OGD (from 303.7 ± 44.76 pg/mg of total protein contents to 259.5 ± 15.44); however, it was not statistically significant. A similar trend was observed in the examined protein contents in rat brains 7 days after HI; however, none of the observed differences was statistically significant (Fig. 10N).

### Influence of neonatal HI on the CNS myelination

**Figure 11:**
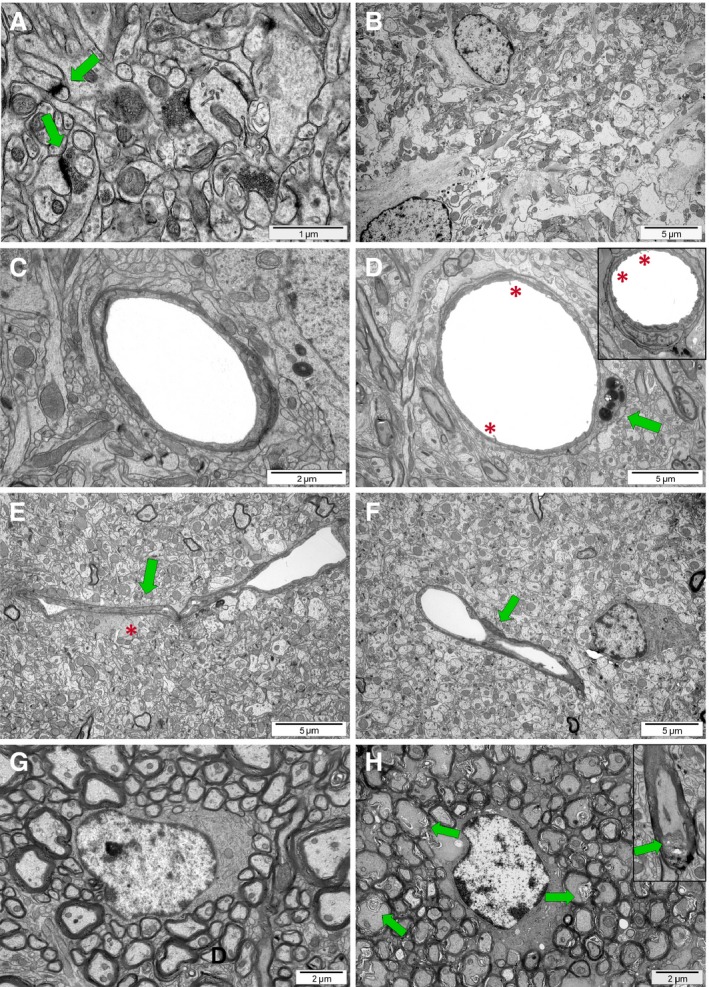
Brain ultrastructure of control and experimental rats 9 weeks (P70) after H‐I insult. (**A**) Characteristic organization of neuropil (easily discernable structural elements like, for example synapses; green arrows) within cerebral cortex of control animals. (**B**) Hydropic, oedematous neuropil in the H‐I brain parenchyma; (**C**) lumen of capillaries and vessels is smooth, with tight intercellular junctions between endothelial cells in controls; (**D**) vascular disorders in brains of H‐I rat: characteristic microvilli on endothelium surface (red asterisk) and a macrophage cell residing in the blood vessel wall (green arrow); (**E**) Collapsing blood vessel (green arrow) in the oedematous neuropil (red asterisk) in hippocampus of H‐I animals; (**F**) Bridging vessel (green arrows) in H‐I rats indicating an ongoing neovascularization; (**G**) compacted myelin enwrapping axons within striatum of control rats; (**H**) malformed myelin sheaths (green arrows) with splitting lamellae surrounding the oligodendroglial cell in striatum of H‐I rats.

The end‐point of oligodendrocyte maturation and their crucial function is the efficient myelination of axons within the developing CNS. The accurateness of the process could be assessed only during an *in vivo* study; therefore, several weeks after the HI insult, the brains of the experimental rats were isolated and prepared for electron microscopic studies (Fig. 1). As the process of myelinating the developing CNS is advanced to a different degree depending on the region of the brain, different brain sections were subjected to an ultrastructural analysis. While in the control rats, neuropil was characterized by presence of typical and easily discernable structural elements (Fig. 11A), analysis of the brain parenchyma revealed areas of oedematous neuropil in rats which experienced the hypoxic‐ischaemic insult (Fig. 11B). Detailed examination of particular regions of the brain revealed also other symptoms of former or still ongoing inflammatory processes within the brains of HI rats. In control brains, lumen of capillaries and vessels is smooth with tight intracellular junctions between endothelial cells (Fig. 11C). There were also no bridging vessels present in any of the analysed regions in the intact nervous tissue. Contrary, the characteristic microvilli on the endothelium surface were found in brains of injured rats, and the microphages residing in the blood vessel wall were present indicating a temporal disruption of its integrity (Fig. 11D). Additionally, the oedema and collapsing vessels could be observed (Fig. 11E). Interestingly, the bridging vessels were frequently present within the brains of animals which experienced perinatal asphyxia, pointing to the still ongoing vascularization. (Fig. 11F). Detailed analysis revealed that the newly forming, bridging vessels accounted for 4% in the cerebral cortex, 2% in the hippocampus and approximately 1.5% in the corpus callosum. In the performed study, no bridging vessels were found in the striatum.

To evaluate the efficiency of myelination, the axon ensheathment was analysed. While in controls, only 1–3% of nervous fibres were surrounded by the uncompacted myelin (Fig. 11G), the malformed myelin sheaths were present more often in hypoxic‐ischaemic brains. Accordingly, 3% of axons were ensheathed by splitting myelin lamellae in the cerebral cortex, 3.5% in corpus callosum, 3–42% in hippocampus (strongly depending on individual animal, the differences were observed even among the littermates) and as much as 41.8% in striatum (Fig. 11H).

## Discussion

The process of active gliogenesis follows derivation of neurons during development of the nervous tissue, and it peaks during the perinatal period in humans 29. The emerging progenitors which will give rise to cells forming the myelin sheath around the CNS axons are known to be extremely sensitive to external stimuli, which may highly influence cell differentiation 30. Generating mature oligodendrocytes capable of myelinogenesis is a complex process comprising a few overlapping stages in which subsequently progenitors, immature cells and finally oligodendrocytes with myelinating potential could be distinguished and described by their characteristic morphology and the expression of the stage‐specific markers. Thus oligodendrocyte differentiation is orchestrated by numerous factors present locally and influencing each stage either by direct contact with the neighbouring cells or in a paracrine manner 31, 32, 33, 34. Supplementing the nervous tissue with the required physiological compounds promoting cell survival (like for instance SHH, PDGF‐AA, FGF‐2) and differentiation (*e.g*. TH3, IGF‐1) seems to be crucial for proper oligodendrocyte maturation 35, 36, 37.

In this context, temporal limitation of trophic support and the resulting alterations in tissue homoeostasis (caused by a perinatal hypoxic‐ischaemic insult occurring to pre‐term infants or due to complications in labour) are thought to significantly contribute to a development of the resulting leukodystrophies, a leading cause of neurodevelopmental disabilities in the affected children 12, 38, 39, 40, 41. Therefore, more in‐depth knowledge of the subject is necessary to be able to describe the influence of temporary HI on the maturation of oligodendrocytes and their ability to efficiently myelinate the developing CNS. To address the issue, we have employed a rat animal model of perinatal asphyxia to mimic the mechanisms developing after the insults occurring to neonatal children. According to the recently published data, the 7^th^ post‐natal day (P7) in rodents is the most relevant to the intense gliogenesis and progress in oligodendrocyte maturation in human neonates 42, 43, 44; thus, the P7 rats were subjected to a temporary hypoxia‐ischaemia with the aim of examining whether and eventually how the insult affects the survival, proliferation and the differentiation of oligodendrocytes.

Taking into consideration that neonatal HI might affect various structures of the brain to a different degree 43, 45, 46, three regions of the brain coronal sections were examined during the performed study: the hippocampus, the striatum and finally the cerebral cortex. Those anatomical structures are commonly used to analyse oligodendrocyte differentiation in various animal models 47, 48, 49. The examination was performed 2 weeks after the HI insult to examine a progress in oligodendrocyte maturation. Additionally, to assess the degree of MMP‐2/MMP‐9 (gelatinases) involvement in oligodendrocyte maturation *in vivo*, the double‐labelling of oligodendrocytes and active forms of the investigated metalloproteinases was performed. An analysis of colocalization of intracellular active forms of MMP‐2/MMP‐9 and maturating oligodendrocytes was carried out to address the question whether perinatal ischaemia‐hypoxia exerts any influence on the expression of gelatinases in response to the insult.

The very first observation points to a significant down‐regulation of the progenitor cells expressing gelatinases in rat hippocampus. However, when the cells were counted and calculated *versus* the total fraction of the MMPs‐expressing cells in the brain, a significant decrease in NG2^+^/MMPs^+^ cells was observed in the cerebral cortex, indicating a decrease in the pool of oligodendrocyte progenitors within this brain section. A developing brain is known to possess a considerable ability to compensate for pre‐ and perinatally acquired focal lesions. On one hand, neuroreparative mechanisms seem to be much more effective than in the adult brain, which could be attributed to the intensive neurogenic and gliogenic processes characteristic for early periods of ontogenesis. On the other hand, due to still existing connections between both hemispheres, the lesion in the ipsilateral hemisphere might also trigger a cellular response and reorganizational changes at the hemisphere which is contralateral to the injury 50, 51. The observed brain plasticity is thought to be a keystone of the compensatory mechanism allowing to eliminate or at least to diminish the fatal consequences of the experienced insult 52, 53. Analysis of the expression of MMP‐2/MMP‐9 in oligodendroglia‐biased cells after the hypoxic‐ischaemic injury in both the *in vivo* and *in vitro* models revealed the presence of active gelatinases in cells at different stages of maturation process. According to our observation, however, there was no significant increase in an endogenous activity of those enzymes after the HI episode. Accordingly, 2 weeks after HI the differentiating GalC‐positive cells with gelatinase activity were present, although the cells were rarely found. Thus, the progress in oligodendroglial maturation was estimated basing on morphology of the cells. This approach allowed us to speculate about alteration in oligodendrocyte differentiation (especially slowdown of maturation process) after neonatal hypoxic‐ischaemic insult.

To verify this presumption, more precise examination was performed on hippocampal organotypic slices. Culturing slices *ex vivo* accelerates processes occurred within the tissue‐like cell maturation and senescence. Thanks to the applied model, mature oligodendroglial phenotypes were visualized several days after temporal deprivation of glucose and oxygen, mimicking *ex vivo* the HI insult. Moreover, the processes of cell survival, their proliferation and differentiation of newly formed OPCs could be followed up to acquire by the cells the ability to express myelin components like myelin basic proteins (MBPs) and proteolipid protein (PLP) 31, 54, 55, 56. Accordingly, a reduction in OPC number in response to HI was confirmed. The smears of BrdU tracker infrequently visible after OGD procedure might suggest that some of the newly generated cells are not able to survive in the tissue microenvironment influenced by the insult. As OGD strongly affects neurons, the compounds released by the cells in response to stress are supposed to disturb local homoeostasis and subsequently modulate the ability of newborn progenitors to survive and differentiate 57, 58, 59. Nonetheless, the progress in differentiation of new oligodendrocytes towards O4‐antigen‐positive stage during 7 DIV was confirmed.

The progress in oligodendroglial maturation and its end‐point manifested by the cell ability to myelinogenesis was followed by the electron microscopic examination of the formed myelin sheaths. As first few post‐natal weeks in rodents is characterized by the intense development of CNS and actually, it is a window period for the activation of the endogenous neuroreparative mechanisms, the anatomical analysis was performed 7 weeks after HI. Taking into account that ensheathment of axons in particular brain structures occurs at the restricted periods of ontogenesis 60, 61, 62, the progress in myelination was examined in corpus callosum, hippocampus, striatum and cerebral cortex. Our findings revealed that while there were relatively large sections within each of the examined region characterized by correctly formed myelin sheaths in affected animals, the focal regions of uncompact myelin were frequently found, especially in hippocampus and in striatum of the affected animals. These observations suggests that either at least the fraction of the survived oligodendrocyte progenitors are able to complete the maturation process and efficiently myelinate axons or compensative mechanisms are initiated to produce myelin by the newly generated oligodendrocytes which replace the OPCs depleted by the insult. Our studies aimed at following the fate of both the OPCs which arisen during the normal ontogenesis, as well as those formed after the insult suggest that each scenario seems to be probable, at least in the investigated experimental model. The recognized focal alterations in myelin structure correlate with the observations of relatively large areas of nervous tissue locally depleted from the mature oligodendrocytes 2 weeks after the insults. Taking into consideration the locally pronounced symptoms of inflammatory process, like oedema and increased number of microglia, it could be supposed that inflammation modulates *in situ* the OPC survival and maturation. Nonetheless, the axon ensheathing is carried out after the insult resulting in a myelin formation, although many of the formed myelin sheaths are characterized by uncompacted and splitting lamellae.

The neurorepair after brain injury is probably promoted also by the increased angiogenesis observed at the ultrastructural level as capillaries and vessel bridging, supporting a reestablishment of functional microvasculature 63, 64.

The ability of developing brain to combat developmental hypomyelination may be also explained by the hypothesis that OPCs are generated in excess during ontogenesis and remain in their undifferentiated form in CNS parenchyma through the lifespan 65, 66 until being mobilized in response to pathological signals to contribute to neurorestorative processes 67, 68, 69, 70. On one hand, this presumption might explain the observation that the number of mature oligodendrocytes is often reported to be normal in spite of the decreased number of progenitors after various insults 71, 72 On the other hand, previous reports described the reduced oligodendrocyte number and deficient myelination as a result of perinatal inflammation 57, 73, 74, 75, 76.

Our study involving experiments on *ex vivo* and *in vivo* models of perinatal asphyxia followed by the detailed ultrastructural examination of the rat brains several weeks after experiencing the HI insult also indicates that the major impediments for oligodendrocyte survival and differentiation might be the inflammatory process. Even in the hippocampal slices, OGD leads to massive proliferation and anti‐inflammatory response of microglial cells 8, 77, 78, which are known also to influence oligodendrocyte differentiation (for review see *e.g*. ref. 79). The present study reveals the local neuropil oedema and indicated the interruption of blood–brain barrier in the past by the alteration in the seamless of the blood vessel walls and the sporadic presence of the macrophages in their vicinity. The ability of OPCs and differentiating oligodendrocytes to overcome the effects of local inflammation seems to be crucial for preventing CNS hypomyelination, and efficiency of this straggle in an unfavourable microenvironment might be reflected by the regional differences in survival and maturation of the cells 80, 81, 82.

Importantly, oligodendrocyte progenitors are able to secrete factors modifying local microenvironment and neighbouring cell response thus potentially contributing to initiation of the endogenous restorative processes 8, 83, 84. In this context, the increased rate of OPC proliferation in response to perinatal ischaemia might be beneficial in a dual way: as helping in combating the inflammation and increasing the chance for proper CNS myelination.

## Conflict of interest

The authors declare that they have no competing interests.
